# Evaluation of nutritional interventions and their impact on metabolic syndrome risk markers in older adults

**DOI:** 10.3389/fnut.2026.1795035

**Published:** 2026-03-05

**Authors:** Lijuan Zhou, Haodong Liu, Yanjia Ma

**Affiliations:** 1 School of Nursing, Shangqiu Medical College, Shangqiu, Henan, China; 2 Department of Emergency Medicine, The First Affiliated Hospital of Zhengzhou University, Zhengzhou, China; 3 Department of Hepatobiliary Surgery, Henan Provincial People's Hospital, Zhengzhou, China

**Keywords:** cardio-metabolic risk, inflammation, metabolic syndrome, nutritional interventions, retrospective study

## Abstract

**Background:**

Metabolic syndrome (MetS) is extremely common in the elderly and strongly increases the chances of cardiovascular disease, diabetes with a type 2, and functional impairment. Although the positive effects of certain dietary interventions have been proved by controlled trials, little evidence has been provided concerning the usefulness of nutritional strategies in the ordinary clinical settings among the elderly population.

**Aim:**

The aim of the study was to assess the effects of documented nutritional intervention on the metabolic syndrome risk markers in older adults based on real-world clinical data.

**Methods:**

The retrospective analysis of electronic medical records of 1,300 adults aged ≥ 60 years with MetS, who received treatment in January 2019–December 2024 in tertiary hospitals and their affiliated outpatient clinics, was conducted. Exposures to nutrition were Mediterranean-style diets, DASH-type diets, low-carbohydrate and high-fiber counseling, calorie limited diets, Omega-3 and vitamin D, and combined interventions. The alterations in the anthropometric parameters, glycemic parameters, lipid parameters, blood pressure parameters, inflammatory/oxidative stress parameters, nutritional intake and MetS status were evaluated. Multivariate logistic regression was used to establish predictors of MetS improvement.

**Results:**

The findings were significant in all the MetS components. Body weight (−2.9 kg), waist circumference (−3.8 cm), visceral fat index (−1.3), fasting glucose (−7.9 mg/dL), HbA1c level (−0.3%), HOMA-IR (−0.7), triglycerides (−24.9 mg/dL), LDL cholesterol (−11.8 mg/dL), systolic blood pressure (−7.7mmH). There was a significant increase in diet quality scores and also the increase in fiber, omega-3, vitamin D, potassium, and magnesium dietary consumption and decrease in sodium, dietary consumption. All in all, 41.4% of the respondents had partial or total MetS resolution. Dietary style of Mediterranean origin, fiber consumption ≥ 25 g/day, ≥ 5% weight loss, omega-3 supplementation, vitamin D deficiency, low sodium consumption, and exercise alone forecasted MetS improvement.

**Conclusion:**

Nutritional interventions are linked to significant changes in metabolic, inflammatory, and cardiovascular risk factors in older adults with MetS in their routine clinical practice. These observations favor the position of real-life dietary interventions as good measures to counteract MetS and enhance healthier aging.

## 1 Introduction

Metabolic syndrome (MetS) is a combination of interrelated cardiometabolic dysfunction such as central obesity, insulin resistance, dyslipidemia, and hypertension which together increase the risk of type 2 diabetes, cardiovascular disease, functional impairment and early mortality. Its rates are particularly high in old age as body composition, mitochondrial activity, chronic low-grade inflammation, and hormonal imbalance are gradually transformed. The elderly population is especially susceptible due to age-related sarcopenia and decreased physical activity combined with a distortion of nutrient metabolism in combination with the long-term exposure to diets as a metabolic risk factor. Current epidemiological data highlights that the occurrence of MetS in adulthood should not be seen as a result of caloric overconsumption, but a result of decades-long nutritional and lifestyle choices, and biological modification of age-related processes. Nutritional determinants are key determinants of the onset and the course of MetS among the aged. An examination of Trinity Ulster Department of Agriculture (TUDA) cohort revealed that dietary quality, micronutrient status, and macronutrient composition positively relate to MetS trajectories over the years, and the deficiency in B-vitamins, improper protein distribution, and the under arching dietary patterns help increase metabolic profiles of elderly individuals (1). The results given imply that metabolic dysregulation associated with nutrition is not fixed at older age, so there is still an opportunity to alter the progression of disease through specific nutritional treatment, even in advanced age. On the same note, population-based statistics in Iran reveal that unhealthy lifestyle and dietary habits, which involve consuming large amounts of refined carbohydrates and low levels of foods rich in nutrients, are uniquely linked with MetS among the elderly, which demonstrates the universal applicability of diet-related metabolic risk factors to aging populations worldwide (2).

Nutritional interventions have a mechanistic effect on the MetS risk markers by affecting several biological pathways interacting with each other. The surplus energy consumption and low nutritional quality of diets favor visceral fats which is an endocrine gland that synthesizes pro-inflammatory cytokines like interleukin-6 and tumor necrosis factor-a, which disrupts insulin signaling and endothelial activity. Diets high in bioactive compounds and especially the polyphenols have been found to fight these processes by decreasing the oxidative stress, enhancing the efficiency of the mitochondria, and regulating the inflammatory pathways. Intervention trials with extended follow-ups that included the intake of higher amounts of polyphenols in addition to promotion of physical activity showed long-term changes in oxidative and inflammatory phenotypes in patients with MetS, which validates the idea that certain dietary ingredients have effects beyond the impact of weight loss (3). These mechanical understandings are consistent with more general evidence that chronic inflammation, redox disequilibrium, gut microbiota changes, and dysregulated lipid and glucose metabolism are pathophysiological mechanisms behind MetS (4).

Weight management continues to be one of the main pillars of the prevention and therapeutic approach of MetS, but its use in the elderly has been questioned because of the risks of muscle wasting and frailty. The recent evidence has shown that purposefully planned dietary interventions with resulting small, but deliberate weight loss can positively influence the insulin sensitivity, lipid profiles, and blood pressure of older adults provided that the intervention is properly planned (5). The mediations of these benefits are achieved through a decrease in ectopic fat deposition, an increase in the adipokines, and an increase in metabolic flexibility. Notably, the composition of diets also seems to be as conditioning as the weight loss itself; in fact, dietary low-carbs approaches have been linked to the improvement of triglycerides, HDL cholesterol, as well as glycemic regulation, but there seems to be a lot of heterogeneity in the responses, and this may depend on an individual metabolic phenotype and age-related factors (6).

In addition to the single-nutrient or single-modality intervention options, there is increasing evidence to support the effectiveness of lifestyle strategies in general. The systematic reviews and network meta-analyses show that multi-modal interventions with dietary modification, physical activities, and behavioral support are more effective than nutritionally only interventions or exercise-only interventions to reverse MetS and even to enhance its individual components (7). The data of the randomized clinical trials also indicate that long-term remission of MetS can be provided under sustained lifestyle interventions, and the adherence and sustainability of interventions are essential (8). The results are especially applicable to older age groups, whereby cumulative lifestyle exposures and established practices may necessitate complex interventions in order to get significant metabolic changes. Other advantages of targeted diet therapy, such as probiotic supplement, have been shown in older adults with MetS, and the systemic effects of nutritional interventions on older patients with the condition have been extended beyond conventional metabolic parameters to other domains, such as risk of sleep apnea and quality of life, which makes the idea of targeted diet therapy in older adults even more significant (9).

Despite substantial progress, important gaps remain in the literature. Most evidence comes from prospective cohorts or randomized trials with predefined interventions, while data on the real-world effectiveness of nutritional strategies in routine clinical or community settings for older adults are limited. Research has largely focused on short- to medium-term outcomes, with insufficient exploration of how prior or ongoing nutritional interventions affect long-term metabolic syndrome (MetS) risk markers. Moreover, the marked heterogeneity of older adults regarding comorbidities, medication use, functional status, and nutritional risk is often underrepresented, restricting generalizability. Retrospective studies therefore provide a valuable means to examine long-term nutritional exposures and metabolic responses in diverse, real-world populations, complementing findings from controlled trials. Against this backdrop, the present retrospective study aims to evaluate the impact of nutritional interventions on key MetS risk indicators in older adults. By examining changes in anthropometric, glycemic, lipid, and inflammatory parameters in relation to documented dietary strategies, this study seeks to bridge the gap between mechanistic and trial-based evidence and real-life clinical outcomes, and to further clarify the role of nutrition in mitigating MetS risk and progression in aging populations.

## 2 Methodology

### 2.1 Study design and setting

This research was approved by the Ethics Committee of the Nursing School of Shangqiu Medical College, Approval Number: SYLL2025-023. The research team conducted their retrospective observational study by using standard clinical data, which had been collected from elderly patients who received treatment at tertiary hospitals and their associated outpatient facilities. The researchers assessed the connection between recorded nutritional intake and subsequent changes in metabolic syndrome indicators through their analysis of electronic medical records, which documented health information from January 2019 until December 2024. The researchers designed the study according to STROBE guidelines and presented their findings through STROBE reporting standards.

The study screened 1,742 electronic medical records during its research period. The study excluded 442 records because they did not fulfill established criteria which required a confirmed metabolic syndrome diagnosis and complete follow-up information and active cancer and severe kidney or liver disease and ongoing corticosteroid or immunosuppressive treatment and complete primary outcome information. The research team analyzed 1,300 study participants for their final analysis. The manuscript now includes a STROBE-compliant participant flow diagram to enhance understanding of this procedure.

### 2.2 Study population

The study examined 1,300 adults who met the age requirement of 60 years or older. The eligibility criteria required participants to have a metabolic syndrome diagnosis that followed NCEP ATP III standards and to provide both initial and subsequent medical information. The participants needed to show proof that they had received at least one type of nutritional education program, dietary guidance, or nutritional aid throughout their standard medical appointments. The researchers excluded participants who showed evidence of active infectious or inflammatory diseases, who had current cancer, who suffered from terminal kidney or liver disease, who had undergone bariatric surgery, or who had received extended treatment with corticosteroids or immunosuppressive drugs. The research excluded records that lacked essential primary outcome information from their ultimate data analysis.

### 2.3 Nutritional exposure assessment

The researchers used medical and dietary records to evaluate how participants were exposed to nutritional elements. The researchers used a Mediterranean-style diet, DASH-type diet, low-carbohydrate diet, high-fiber dietary counseling, calorie-restricted diet, omega-3 fatty acid supplementation, and vitamin D supplementation combined with dietary and supplementation methods and regular dietary guidance to classify participants according to their main dietary pattern and supplemental treatment, which they received during follow-up. The routine clinical practice of healthcare professionals created these exposures, which existed in their normal work environment. The study team used follow-up documentation and repeat consultations together with existing dietary records to assess dietary adherence, which they could not measure directly.

The documentation of nutritional interventions originated from multiple sources which included physician clinical notes and registered dietitian consultation records and structured dietary counseling templates and supplement prescription records and patient-reported dietary adherence documented during follow-up visits. The study participants were divided into different groups based on their main recorded eating habits. The study used the primary intervention which appeared in over half of the follow-up sessions to classify multiple recorded interventions. The researchers established dietary pattern exposure categories through operational criteria which defined specific nutrient and food-based targets.

### 2.4 Data collection and variables

The research team followed a standardized protocol for data extraction. The researchers collected demographic information through age and sex variables. The researchers measured anthropometric data by collecting body weight and calculating body mass index (BMI), waist and hip circumference, waist-to-hip ratio, body fat percentage, lean mass, visceral fat index, and mid-arm circumference and neck circumference. Blood pressure assessment involved measuring systolic blood pressure, diastolic blood pressure, pulse pressure, mean arterial pressure, and blood pressure variability measurements.

The biochemical variables measured in the study included fasting plasma glucose levels and glycated hemoglobin (HbA1c), fasting insulin and homeostasis model assessment of insulin resistance (HOMA-IR), postprandial glucose and lipid profile parameters, which included total cholesterol, LDL cholesterol, HDL cholesterol, triglycerides, non-HDL cholesterol, apolipoprotein B, and the metabolic syndrome components. A subset of participants (n = 612) received tests that measured inflammatory and oxidative stress markers that included C-reactive protein, interleukin-6, tumor necrosis factor-*α*, fibrinogen, oxidized LDL, adiponectin, leptin, erythrocyte sedimentation rate, and nitric oxide.

The dietary variables measured in the study included total energy intake and macronutrient distribution, fiber intake, sodium and potassium intake, omega-3 intake and vitamin D levels, and magnesium intake and diet quality scores for a subset of participants who had documented their dietary activities (*n* = 724).

The trained clinical staff conducted anthropometric measurements through the use of calibrated digital scales and standardized stadiometers. Blood pressure was measured using validated automated oscillometric devices after a 5-min seated rest, with the average of two readings recorded. The medical staff collected fasting blood samples between 7:00 and 9:00 a.m. after the patients completed their overnight fast which lasted for at least 8 h. Certified hospital laboratories conducted biochemical analyses through standardized enzymatic methods for metabolic parameters and ELISA-based assays for inflammatory markers. The clarifications provide better methodological transparency which enables researchers to reproduce their research findings.

### 2.5 Outcome measures

The primary outcome was the change in metabolic syndrome status, which researchers assessed by measuring the reduction in MetS diagnostic criteria between baseline and follow-up assessments. The secondary outcomes measured the variations that occurred in individual MetS components that included central obesity, blood pressure, glycemic indices, lipid parameters, and inflammatory markers, together with the changes that happened in dietary intake patterns and nutrient-related biomarkers.

### 2.6 Statistical analysis

Statistics version 8.1 was used to conduct their statistical analyses. The study reported continuous variables using mean values and standard deviation values, while categorical variables were shown through frequency counts and percentage values. The researchers used paired t-tests and Wilcoxon signed-rank tests to analyze continuous data, while categorical data was assessed through chi-square tests to compare baseline measurements with follow-up assessments.

The research used multivariate logistic regression analysis to determine which factors showed independent links to metabolic syndrome improvement. The study assessed eight covariates that included age and sex, initial metabolic syndrome status, weight fluctuations, dietary exposure, supplement usage, sodium consumption, and vitamin D levels, and documented physical activity. The study presented results through odds ratios that included 95% confidence intervals. The research identified statistical significance at the threshold of a two-sided *p*-value less than 0.05.

The researchers selected variables for their multivariable logistic regression models through a process which included both clinical assessment and existing research studies. The researchers assessed multicollinearity through variance inflation factor calculations, while they used the Hosmer–Lemeshow goodness-of-fit test to assess model calibration. The study showed minimal missing data for primary outcomes which were less than 5 percent and the researchers conducted complete-case analysis. The researchers conducted subset analyses, which examined dietary biomarkers and inflammatory markers while they limited their study to participants who had complete data access. The researchers conducted sensitivity analyses to test how well their results would hold under different conditions.

### 2.7 Ethical considerations

This research was approved by the Ethics Committee of the Nursing School of Shangqiu Medical College, Approval Number: SYLL2025-023. The Institutional Review Board approved the study protocol after its complete review process. The study researchers did not require participants to provide written informed consent because they used anonymized medical records for their retrospective research. The study followed all institutional data protection rules while upholding the Declaration of Helsinki ethical standards.

## 3 Results

### 3.1 Baseline demographic and clinical characteristics

The study population included older adults who had multiple metabolic risk factors, which resulted in above-average health problems. The study participants had a mean age of 68.6 years, which showed they belonged to an elderly group who met research criteria for metabolic syndrome studies. Males formed a slight majority of the group at 53.4 percent, while both genders appeared in sufficient numbers to improve study results. The study participants showed an average body mass index of 29.3 kilograms per square meter with central obesity, which increased their risk of developing cardio metabolic conditions according to health standards. The study participants displayed increased blood pressure levels, which included a mean systolic blood pressure of 139.1 mmHg and a mean diastolic blood pressure of 85.1 mmHg, which indicated they had a high chance of developing hypertension. The study results showed poor glycemic control because the participants had fasting plasma glucose levels of 116.8 mg/dL, which matched the criteria for impaired fasting glucose. The study participants showed dyslipidemia through their high triglyceride levels of 181.6 mg/dL and low HDL cholesterol levels of 41.8 mg/dL. The research results established that the group showed typical metabolic syndrome characteristics, which scientists used to determine how nutritional treatments affected their cardio metabolic health results (Table 1).

**Table 1:** Baseline demographic and clinical characteristics of the study population.

Parameter	Mean ± SD / n (%)
Age	68.6 ± 6.8
Male	694 (53.4%)
Female	606 (46.6%)
BMI (kg/m^2^)	29.3 ± 4.9
Waist circumference (cm)	101.2 ± 11.9
Systolic BP (mmHg)	139.1 ± 18.2
Diastolic BP (mmHg)	85.1 ± 10.6
Fasting plasma glucose (mg/dL)	116.8 ± 27.4
Triglycerides (mg/dL)	181.6 ± 52.3
HDL cholesterol (mg/dL)	41.8 ± 10.4

BMI, body mass index; BP, blood pressure; HDL, high-density lipoprotein; SD, standard deviation.

### 3.2 Distribution of documented nutritional exposures

The Mediterranean dietary pattern required people to use olive oil and whole grains and legumes and eat fish regularly while reducing their red meat consumption. The DASH-type diet required people to limit sodium intake while consuming large amounts of fruits and vegetables. Low-carbohydrate diets required people to consume less than 40 percent of their daily calories from carbohydrates. The dietary counseling program required participants to consume at least 25 grams of dietary fiber each day. The definition of calorie-restricted diets required participants to maintain daily energy deficits between 500 and 750 kilocalories. The study defined supplement exposures through documented cases of prescription or recommendation. The researchers divided the study participants into different exposure groups according to their primary method of treatment. Mediterranean-style dietary advice received the highest frequency of exposure documentation, which reached 22.9% because medical research proved its cardio metabolic health advantages for older adults. The DASH-type diet was also commonly recommended (16.5%), consistent with its established role in blood pressure and cardiovascular risk reduction. The two dietary methods, which targeted glycemic control and lipid profile improvement, used low-carbohydrate diets (13.5%) and high-fiber dietary counseling (12.5%) as their main approaches. The study found that 11.8% of participants followed calorie-restricted diets, which researchers used to help people control their weight in a community that showed high rates of excess weight and central obesity. Supplement-based exposures were less frequent, with omega-3 (7.4%) and vitamin D (6.0%) supplementation reflecting adjunctive approaches rather than primary strategies (Table 2).

**Table 2:** Distribution of documented nutritional exposures among participants.

Nutritional exposure	Participants n (%)
Mediterranean-style dietary advice	298 (22.9%)
DASH-type diet	214 (16.5%)
Low-carbohydrate diet	176 (13.5%)
High-fiber dietary counseling	162 (12.5%)
Calorie-restricted diet	154 (11.8%)
Omega-3 supplementation	96 (7.4%)
Vitamin D supplementation	78 (6.0%)
Combined diet + supplements	72 (5.5%)
Usual care / general advice	50 (3.9%)

DASH, Dietary Approaches to Stop Hypertension.

### 3.3 Changes in anthropometric parameters following nutritional exposure

The subjects experienced an average weight reduction of 2.9 kilograms, which resulted in a BMI decrease of 1.1 kilograms per square meter that reached statistical significance through both tests. The waist circumference reduction of 3.8 centimeters, together with the hip circumference reduction of 2.6 centimeters, which both showed statistical significance at *p* < 0.001, resulted in a small yet measurable waist-to-hip ratio decrease of 0.01 at *p* = 0.04. The study found that body fat percentage decreased by 1.8 percent while visceral fat index showed a substantial reduction of 1.3 (*p* < 0.001), which indicates that the body lost primarily dangerous metabolic fat stores. The study detected small but meaningful circumferential decreases at both the mid-arm and neck areas, which indicated that body composition had improved across all body regions. The study found that participants maintained their lean mass at almost the same level as before (−0.2 kg, *p* = 0.19), which indicates that weight loss occurred because of fat mass decrease and not through muscular body weight loss (Table 3).

**Table 3:** Changes in anthropometric parameters following nutritional exposure.

Parameter	Baseline	Follow-up	Mean change	*p*-value
Body weight (kg)	79.1 ± 13.4	76.2 ± 13.1	−2.9	<0.001
BMI (kg/m^2^)	29.3 ± 4.9	28.2 ± 4.8	−1.1	<0.001
Waist circumference (cm)	101.2 ± 11.9	97.4 ± 11.6	−3.8	<0.001
Hip circumference (cm)	104.7 ± 10.3	102.1 ± 10.1	−2.6	<0.001
Waist-to-hip ratio	0.97 ± 0.07	0.96 ± 0.07	−0.01	0.04
Body fat (%)	35.2 ± 7.4	33.4 ± 7.2	−1.8	<0.001
Lean mass (kg)	47.6 ± 8.7	47.4 ± 8.5	−0.2	0.19
Visceral fat index	13.4 ± 3.9	12.1 ± 3.7	−1.3	<0.001
Mid-arm circumference (cm)	31.8 ± 4.2	31.3 ± 4.1	−0.5	0.03
Neck circumference (cm)	39.5 ± 3.9	38.7 ± 3.8	−0.8	0.01

BMI, body mass index; SD, standard deviation.

### 3.4 Changes in glycemic parameters following nutritional exposure

**Figure 1:**
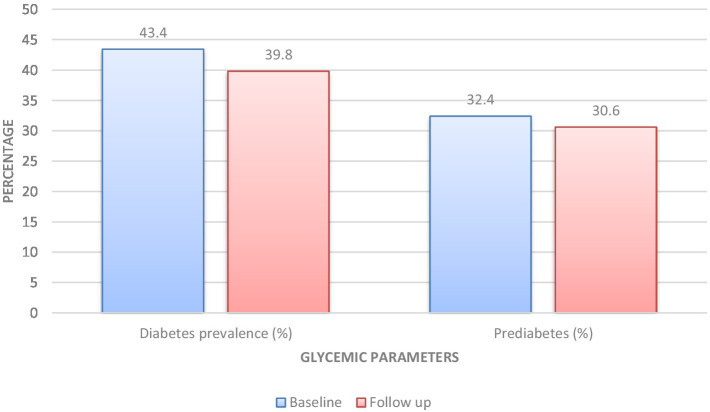
Changes in glycemic parameters over follow-up.

The study showed that fasting glucose levels dropped by 7.9 mg/dL and postprandial glucose levels decreased by 14.8 mg/dL (*p* < 0.001 for both). The HbA1c test showed a small but statistically significant decrease of −0.3% (*p* < 0.001). Participants experienced decreased fasting insulin levels and HOMA-IR values which dropped by −1.9 μIU/mL and −0.7, respectively, (*p* ≤ 0.002). The insulin sensitivity index showed an increase of 0.4 (*p* = 0.003). Diabetes prevalence decreased by 3.6% (*p* = 0.04), while prediabetes presented a non-significant decline. Glycemic variability had a significant improvement with a decrease of −1.8 (*p* = 0.01). The structured nutritional interventions resulted in improvement for 8.4% of participants who initially met the metabolic syndrome glycemic criterion which produced statistically significant results (*p* < 0.001; Table 4; Figure 1).

**Table 4:** Changes in glycemic parameters over follow-up.

Parameter	Baseline	Follow-up	Mean change	*p*-value
Fasting glucose (mg/dL)	116.8 ± 27.4	108.9 ± 25.6	−7.9	<0.001
HbA1c (%)	6.4 ± 1.0	6.1 ± 0.9	−0.3	<0.001
Fasting insulin (μIU/mL)	15.1 ± 7.6	13.2 ± 7.1	−1.9	0.002
HOMA-IR	4.3 ± 2.6	3.6 ± 2.3	−0.7	<0.001
Postprandial glucose (mg/dL)	171.2 ± 42.1	156.4 ± 39.8	−14.8	<0.001
Glycemic variability	22.1 ± 7.4	20.3 ± 7.1	−1.8	0.01
Insulin sensitivity index	3.5 ± 1.3	3.9 ± 1.4	+0.4	0.003
MetS glycemic criterion met (%)	69.1	60.7	−8.4	<0.001

HbA1c, glycated hemoglobin; HOMA-IR, homeostasis model assessment of insulin resistance; MetS, metabolic syndrome.

### 3.5 Lipid profile changes following nutritional exposure

Total cholesterol levels decreased by 14.4 mg/dL, while LDL cholesterol levels dropped by 11.8 mg/dL, with both results reaching statistical significance at *p* < 0.001. HDL cholesterol levels increased by 3.1 mg/dL. The reduction showed a decrease of 24.9 mg/dL in triglycerides, which included a reduction of the TG/HDL ratio (−0.8, *p* < 0.001) and non-HDL cholesterol (−17.5 mg/dL, *p* < 0.001). The atherogenic indices demonstrated a significant reduction of both the atherogenic index and the small dense LDL percentage, with results of −0.09 and *p* value of 0.002, and results of −5.6 percent and p value of 0.01, respectively. The atherogenic indices demonstrated that cardiovascular risk had decreased. ApoB levels decreased by 10.2 mg/dL (*p* < 0.001), which showed a decrease in atherogenic particles that circulate in the bloodstream. The metabolic syndrome lipid criterion showed an 11.8% decrease in the research group after dietary changes (Table 5).

**Table 5:** Lipid profile changes during follow-up.

Parameter	Baseline	Follow-up	Mean change	*p*-value
Total cholesterol (mg/dL)	216.8 ± 42.7	202.4 ± 40.8	−14.4	<0.001
LDL cholesterol (mg/dL)	137.6 ± 35.9	125.8 ± 34.1	−11.8	<0.001
HDL cholesterol (mg/dL)	41.8 ± 10.4	44.9 ± 10.9	+3.1	<0.001
Triglycerides (mg/dL)	181.6 ± 52.3	156.7 ± 49.6	−24.9	<0.001
Non-HDL cholesterol	175.0 ± 40.1	157.5 ± 38.4	−17.5	<0.001
TG/HDL ratio	4.5 ± 2.2	3.7 ± 2.0	−0.8	<0.001
Atherogenic index	0.58 ± 0.25	0.49 ± 0.23	−0.09	0.002
Small dense LDL (%)	39.4	33.8	−5.6	0.01
ApoB (mg/dL)	109.8 ± 26.9	99.6 ± 25.7	−10.2	<0.001
Lipid MetS criterion met (%)	73.2	61.4	−11.8	<0.001

ApoB, apolipoprotein B; HDL, high-density lipoprotein; LDL, low-density lipoprotein; MetS, metabolic syndrome; TG, triglycerides.

### 3.6 Blood pressure outcomes following nutritional exposure

**Figure 2:**
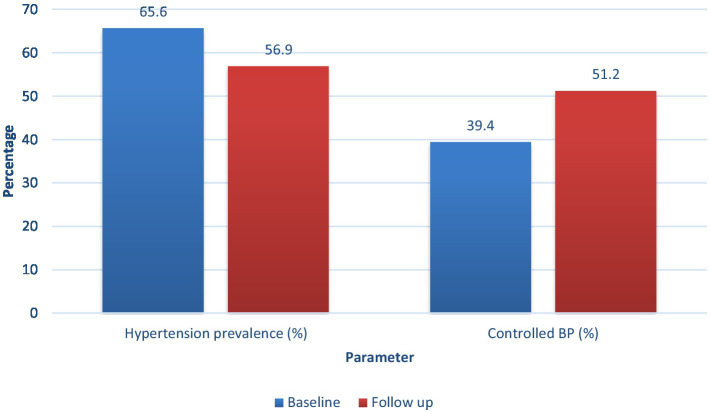
Blood pressure outcomes over follow-up.

Systolic and diastolic blood pressure values decreased by 7.7 mmHg and 4.5 mmHg, respectively (*p* < 0.001 for both), while pulse pressure (−3.2 mmHg, *p* = 0.004) and mean arterial pressure (−5.6 mmHg, *p* < 0.001) showed decreases that resulted in total cardiovascular advantages. Blood pressure variability for systolic and diastolic measurements decreased (−1.6 mmHg, *p* = 0.01; −1.4 mmHg, *p* = 0.02), which indicates that hemodynamic stability had improved. Hypertension prevalence decreased by 8.7% (*p* = 0.002), while the proportion of participants achieving controlled BP increased by 11.8% (*p* < 0.001). Nocturnal blood pressure dipping decreased by 6.8% (*p* = 0.04), which indicated that body processes now control blood pressure throughout the day better than before (Table 6; Figure 2).

**Table 6:** Blood pressure outcomes over follow-up.

Parameter	Baseline	Follow-up	Mean change	*p*-value
Systolic BP (mmHg)	139.1 ± 18.2	131.4 ± 17.6	−7.7	<0.001
Diastolic BP (mmHg)	85.1 ± 10.6	80.6 ± 10.1	−4.5	<0.001
Pulse pressure (mmHg)	54.0 ± 13.2	50.8 ± 12.7	−3.2	0.004
Mean arterial pressure	103.1 ± 13.8	97.5 ± 13.2	−5.6	<0.001
SBP variability	13.4 ± 5.1	11.8 ± 4.9	−1.6	0.01
DBP variability	8.8 ± 3.6	7.4 ± 3.4	−1.4	0.02
Nocturnal dipping (%)	42.8	49.6	+6.8	0.04
BP MetS criterion met (%)	71.2	62.8	−8.4	<0.001

BP, blood pressure; DBP, diastolic blood pressure; MetS, metabolic syndrome; SBP, systolic blood pressure.

### 3.7 Inflammatory and oxidative stress markers

The nutritional interventions brought about major decreases in all body systems that showed signs of inflammation. The tests showed that C-reactive protein dropped 1.1 mg/L (*p* < 0.001) and IL-6 decreased 1.1 pg./mL (*p* = 0.002), and TNF-*α* diminished 0.8 pg./mL (*p* = 0.01), which proved that pro-inflammatory cytokine activity had decreased in the body. The two tests showed significant decreases in both fibrinogen levels and ESR results (−22.6 mg/dL, *p* = 0.01; −2.8 mm/h, *p* = 0.04), which demonstrated a decrease in the body’s inflammatory disease load. Oxidized LDL levels decreased by 7.7 units (*p* = 0.02), indicating that the body experienced a reduction in oxidative stress and a lower potential for atherogenic buildup. The body showed changes in adipokines because adiponectin levels increased by 0.9 μg/mL (*p* = 0.01) and leptin levels decreased by 3.3 ng/mL (*p* = 0.03), which showed better metabolic signaling in the body. Nitric oxide levels increased by 4.3 μmol/L (p = 0.02), which demonstrated better endothelial function in the body (Table 7).

**Table 7:** Inflammatory and oxidative stress markers.

Marker	Baseline	Follow-up	Change	*p*-value
C-reactive protein (mg/L)	4.7 ± 2.5	3.6 ± 2.2	−1.1	<0.001
IL-6 (pg/mL)	6.9 ± 3.8	5.8 ± 3.4	−1.1	0.002
TNF-α (pg/mL)	7.3 ± 3.6	6.5 ± 3.3	−0.8	0.01
Fibrinogen (mg/dL)	381.2 ± 92.4	358.6 ± 88.1	−22.6	0.01
Oxidized LDL	69.1 ± 24.3	61.4 ± 23.1	−7.7	0.02
Adiponectin (μg/mL)	6.5 ± 2.6	7.4 ± 2.8	+0.9	0.01
Leptin (ng/mL)	29.1 ± 12.1	25.8 ± 11.7	−3.3	0.03
ESR (mm/h)	22.9 ± 9.3	20.1 ± 9.1	−2.8	0.04
Nitric oxide	34.3 ± 10.4	38.6 ± 10.9	+4.3	0.02
Inflammation score	3.9 ± 1.4	3.1 ± 1.3	−0.8	<0.001

ESR, erythrocyte sedimentation rate; IL, interleukin; LDL, low-density lipoprotein; TNF, tumor necrosis factor.

### 3.8 Nutrient intake and dietary biomarkers

The nutritional interventions produced a total energy intake decrease of 212 kcal per day, which reached statistical significance at a *p*-value below 0.001. Participants increased their intake of fiber by 5.7 grams per day, which reached statistical significance at a *p*-value below 0.001. Participants increased their intake of monounsaturated fatty acids by 3.5 percent, which reached statistical significance at a *p*-value below 0.001. A dietary pattern shift toward cardioprotective foods, which included 0.4 grams of omega-3 fatty acids. Vitamin D levels increased by 5.5 ng/mL. Magnesium intake increased by 40 mg per day, which the researchers established at a statistically significant level. Sodium intake decreased by 552 mg per day, which reached statistical significance at a p-value below 0.001. Potassium intake increased by 472 mg per day, which reached statistical significance at a p-value below 0.001 (Table 8).

**Table 8:** Nutrient intake and dietary biomarkers.

Parameter	Baseline	Follow-up	Change	*p*-value
Energy intake (kcal/day)	2,106 ± 452	1894 ± 426	−212	<0.001
Fiber intake (g/day)	18.9 ± 7.2	24.6 ± 7.9	+5.7	<0.001
Saturated fat (%)	12.6 ± 4.1	9.8 ± 3.6	−2.8	<0.001
MUFA (%)	13.3 ± 3.9	16.8 ± 4.4	+3.5	<0.001
Omega-3 intake (g/day)	0.7 ± 0.4	1.1 ± 0.6	+0.4	0.002
Vitamin D (ng/mL)	22.1 ± 9.6	27.6 ± 10.4	+5.5	<0.001
Magnesium (mg/day)	289 ± 86	329 ± 94	+40	0.01
Sodium (mg/day)	3,398 ± 762	2,846 ± 711	−552	<0.001
Potassium (mg/day)	2,514 ± 584	2,986 ± 642	+472	<0.001
Diet quality score	53.4 ± 12.9	66.2 ± 14.7	+12.8	<0.001

MUFA, monounsaturated fatty acids.

### 3.9 Changes in metabolic syndrome status

**Figure 3:**
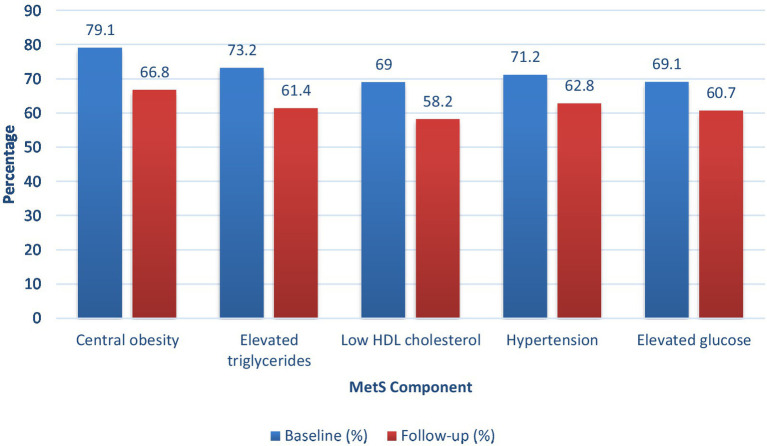
Changes in metabolic syndrome status from baseline to follow-up.

All individual MetS components showed significant progress throughout the study period. The study demonstrated meaningful progress, as central obesity decreased by 12.3% among participants, elevated triglycerides dropped by 11.8%, and low HDL cholesterol levels fell by 10.8%. The two health conditions showed 8.4% reduction. A 16.5% decrease in participants who met three or more MetS criteria and an 11.6% decline in participants who met four or more criteria, which resulted in lower metabolic risk levels. 12.8% of participants achieved complete MetS resolution, while 28.6% achieved partial resolution, resulting in 41% of participants showing significant clinical improvement. Structured nutritional interventions successfully reduced persistent MetS by 13.3%, which proves their power to reverse or lessen metabolic syndrome among older adults (Table 9; Figure 3).

**Table 9:** Changes in metabolic syndrome status from baseline to follow-up.

MetS component	Baseline (%)	Follow-up (%)	Absolute change
≥3 MetS criteria	65.4	48.9	−16.5
≥4 MetS criteria	39.2	27.6	−11.6
Complete MetS resolution	0	12.8	+12.8
Partial resolution	0	28.6	+28.6
Persistent MetS	65.4	52.1	−13.3

HDL, high-density lipoprotein; MetS, metabolic syndrome.

### 3.10 Predictors of metabolic syndrome improvement

The Mediterranean-style diet was associated with a 27% higher improvement in MetS (OR 0.73, 95% CI 0.60–0.88, *p* = 0.002), providing cardiometabolic advantages. The study found that high fiber intake (≥25 g/day) and weight loss of at least 5 percent were both strong independent predictors, decreasing the risk of ongoing MetS by 24 percent and 35 percent, respectively (*p* ≤ 0.006 and *p* < 0.001). The study found that Omega-3 supplementation, vitamin D sufficiency, and reduced sodium intake improved outcomes (OR 0.80–0.84, *p* ≤ 0.03), which demonstrated that targeted nutrient interventions improve health outcomes. Physical activity (OR 0.77, *p* = 0.01) decreased the chance of experiencing adverse health outcomes. Advanced age and higher MetS baseline severity created a greater chance for people to maintain MetS (OR 1.06 and 1.48, *p* ≤ 0.04) while male sex displayed a trend that did not reach statistical significance (Table 10).

**Table 10:** Multivariate logistic regression analysis for predictors of MetS improvement.

Predictor	β	SE	OR (95% CI)	*p*-value
Mediterranean-style diet	−0.31	0.10	0.73 (0.60–0.88)	0.002
Fiber intake ≥25 g/day	−0.28	0.09	0.76 (0.63–0.92)	0.006
Weight loss ≥5%	−0.43	0.11	0.65 (0.52–0.81)	<0.001
Omega-3 supplementation	−0.22	0.08	0.80 (0.68–0.94)	0.01
Vitamin D sufficiency	−0.17	0.07	0.84 (0.73–0.97)	0.02
Reduced sodium intake	−0.19	0.08	0.83 (0.71–0.97)	0.03
Physical activity (adjusted)	−0.26	0.09	0.77 (0.64–0.93)	0.01
Age	0.06	0.03	1.06 (1.01–1.12)	0.04
Male sex	0.09	0.05	1.09 (0.99–1.21)	0.07
Baseline MetS severity	0.39	0.10	1.48 (1.22–1.79)	<0.001

CI, confidence interval; MetS, metabolic syndrome; OR, odds ratio; SE, standard error.

## 4 Discussion

The current retrospective study shows that elderly persons with known metabolic syndrome show significant changes in anthropometric, glycemic, and lipid variables after reported nutritional interventions despite the heterogeneity of real-life populations. The study revealed that at the baseline, the participants were characterized by typical manifestations of the metabolic syndrome such as central obesity, hypertension, dysglycemia, and atherogenic dyslipidemia, which ensured that the cohort was a high-risk older population to assess the cardiometabolic reactions to dietary interventions. These baseline features are congruent with previous observational data on finding that older adults often manifest clumped metabolic pathologies, which are indicative of prolonged exposure to inefficient dietary habits and lifestyle choices. The nutritional exposure distributions in this study are in line with the modern clinical practice, which is dominated by the Mediterranean-style and DASH-type nutritional recommendations, which in turn are dominated by low-carbohydrate, high-fiber and calorie-restricted dietary prescriptions. This is consistent with the community and intervention studies evidence that indexes of dietary quality and nutrition education have a close relationship with the prevalence and severity of metabolic syndrome. Grant found out that the more adherence to the Healthy Eating Index-2015, the worse the elements of metabolic syndrome, which is why the general quality of diet is more important than the specific manipulation of nutrients (10). Likewise, interventions with Bangladeshi adults based on nutrition education resulted in considerable changes in the waist circumference, blood pressure, and lipid parameters, which makes it clear that pragmatic dietary interventions with a focus on education can also provide certain metabolic outcomes that would not be achieved in a strictly regulated trial (11). Multimodal place-based nutritional interventions at the workplace have also provided positive impacts on glucose and lipid metabolism which also confirms the fact that various dietary measures, when adhered to, can enhance cardio-metabolic health in various populations (12).

The observed weight loss, body mass (BMI), waist circumference and visceral fat reduction in this study is especially interesting, considering the age of the subjects (advanced age) and the maintenance of the lean mass. One of the primary causes of metabolic syndrome is central adiposity which contributes to insulin resistance and chronic low-grade inflammation. The resultant reduction in the waist circumference is in line with evidence presented in meta-analyses that lifestyle-based interventions may considerably decrease abdominal obesity and inflammatory load in patients with metabolic syndrome (13). Notably, the fact that lean mass is maintained is in line with the growing body of evidence highlighting the quality of body composition as an independent variable of metabolic health as opposed to weight loss. Even though the mechanistic literature puts more emphasis on younger groups, research has shown that fat mass and fat viscerality reductions are strongly correlated with increased metabolic flexibility and substrate oxidation, irrespective of total body mass (14). Furthermore, the evidence of a possible indirect effect of dietary interventions on body composition via gut microbiome regulation is also increasing, and may be particularly useful in aging individuals (15).

Another important result of the study is the improvement of glycemic parameters. Considerable fasting and postprandial glucose, HbA1c, insulin levels, and HOMA-IR reductions, as well as increases in insulin sensitivity, confirm the fact that the nutritional interventions helped to address the primary, central pathological feature of metabolic syndrome, which is insulin resistance. These findings support the fact that dietary interventions like the DASH diet have the potential to enhance insulin resistance and lipid accumulation indices in patients with metabolic syndrome, which is probably achieved by generating synergistic effects on energy balance, dietary fiber intake, and anti-inflammatory nutrient profiles (16). Extensive reviews have further noted that dietary interventions that focus on whole foods, low refined carbohydrates, and healthier fat aims have positive implications on the process of glucose metabolism by enhancing the insulin signaling and lowering the ectopic fat deposition (17). As in the case of older adults, it has been linked to healthier dietary patterns that further reduce the accumulation of metabolic comorbidities, which may indicate that those gains in glycemic control have extended implications on multimorbidity curves in aging populations (18). The clinical relevance of the changes is further shown by the observed decrease in the percentage of participants that fulfilled the metabolic syndrome glycemic criterion.

The positive lipid profile shifts that were found in this paper also resound the cardio-protective effects of nutritional interventions in elderly people. The decrease in total cholesterol, LDL cholesterol, triglycerides, non-HDL cholesterol, ApoB and atherogenic index and increment in HDL cholesterol show that all lipid processes have been improved. The findings align with randomized evidence that a change in the quality of dietary fats, including the increase in the consumption of unsaturated fats and whole-food sources like nuts, may have a large effect on the lipoprotein profiles of individuals with a high cardio-metabolic risk (19). Retrospective studies have also shown that increased quality of dietary fats is linked to reduced atherogenic lipid markers underpinning the significance of dietary composition in addition to caloric restriction (20). Further, cross-sectional evidence of populations in old age shows that healthier diets, especially active lifestyles coupled, are linked to enhanced glycolipemic metabolism and less inflammatory burden, which promotes the value-added metabolic effects of the current cohort (21). This decrease in the ratio of those participants who passed the metabolic syndrome lipid requirement indicates that the stated biochemical gains might be converted into significant decreases in cardiovascular risk.

The notable changes in systolic and diastolic blood pressure, pulse pressure, mean arterial pressure and change in blood pressure variability demonstrate that nutritional interventions have clinically significant antihypertensive effects in older adults with metabolic syndrome. It is specifically important that the extent of systolic blood pressure reduction (−7.7 mmHg) has been shown to significantly lower cardiovascular morbidity and mortality rates even in older adults whenever reduced by even small degrees. These results are supported by recent network meta-analysis evidence that shows that dietary patterns include the Mediterranean, DASH, and anti-inflammatory diets are some of the most effective non-pharmacological interventions to achieve better blood pressure control in metabolic syndrome patients (22). Meta-analytic evidence further shows that the compliance with a Mediterranean food pattern results in meaningful decreases in systolic and diastolic blood pressure, glycemic and lipid parameters, which contributes to the multidimensional cardio-metabolic advantages of the given strategy (23). Also, the use of randomized trials comparing intermittent fasting, portfolio-style, and anti-inflammatory diets have shown a better blood pressure regulation and vascular functioning in pre-diabetic and metabolically vulnerable groups, which prove the importance of dietary timing and composition in the regulation of hemodynamics (24). These findings are applicable to the real-world population of the elderly as the present study has shown a reduction in hypertension and an increase in the state of controlled blood pressure as a result of structured nutritional guidance in addition to pharmacotherapy.

The extensive decrease of inflammatory and oxidative stress indicators in this experiment shows the anti-inflammatory effects of nutritional interventions in older adults with metabolic syndrome. Marked reductions in CRP, IL-6, TNF-a, fibrinogen and ESR indicate that the chronic low-grade inflammation underlying insulin resistance, endothelial dysfunction and atherogenesis is inhibited. These findings are consistent with the systematic evidence suggesting the links between the diets characterized by high concentrations of polyphenols and bioactive compounds, especially anthocyanins, with the decreases in the inflammatory markers and the mitigation of the risk factors of the metabolic syndrome (25). Findings of meta-analyses also support the idea that dietary interventions may remarkably reduce the circulant levels of inflammatory biomarkers in patients with metabolic syndrome, regardless of weight reduction, and that the quality of food and bioactive nutrients might be overridden to affect immune-metabolic processes (26). The oxidized LDL levels reduced and the salutary changes in adipokines such as higher levels of adiponectin and lower levels of leptin suggest that there was an improvement in oxidative homeostasis and adipose tissue signaling. The evidence that helped to support these findings is that supplementation with natural compounds rich in antioxidants has the potential to alleviate oxidative stress and inflammatory burden and, consequently, improve cardiometabolic health (27). The resultant upsurge in nitric oxide concentration is also an indication of increased endothelial activity, which supports the association between anti-inflammatory dietary habits and vascular performance in older adults.

The reported positive changes in nutrient intake and dietary biomarkers have a mechanistic basis of the positive cardiometabolic outcomes among this cohort. Declines in total energy intake, saturated fat intake, and sodium intake and increases in the status of dietary fiber, monounsaturated fatty acids, omega-3 fatty acids, potassium, magnesium, and vitamin D status represent a change in favor of cardioprotective dietary patterns. Meta-analytic data has shown that the adoption of the Mediterranean dietary pattern is linked to weight and glycemic control and lipid profile and cardiovascular risk factors improvement, which are largely mediated by increased fiber and unsaturated fat and decreased sodium intake (28). The use of dietary patterns based on the idea of dietary patterns following a non-communicable disease-friendly diet is further shown by network meta-analyses based on the idea that such dietary patterns are more effective in promoting the improvements in non-communicable disease biomarkers, such as those associated with metabolic syndrome (29). These caloric decrease and diet quality improvements are also aligned with the existing research obtaining that systematic dietary interventions, such as intermittent fasting plans, can result in desirable body composition and cardiometabolic results in case implemented properly (30).

The significant decrease in the occurrence and the severity of metabolic syndrome experienced in this study highlights the long-term effects of nutritional interventions on cluster cardiometabolic risk. The decrease in the individual MetS factors- central obesity, dyslipidemia, hypertension and hyperglycemia was translated into significant reduction in the number of participants satisfying three or more of the diagnostic criteria with significant percentage being completely and partially resolved MetS. This is in line with the evidence of systematic reviews and network meta-analyses that show that multi-component lifestyle interventions, especially dietary change-based interventions, are more effective in reversing metabolic syndrome than single-modality interventions (7). Comparative studies also suggest that the Mediterranean-style dietary patterns are better than other therapeutic interventions in enhancing the MetS elements and lowering the overall cardio-metabolic risk (31). Interventions based on nutrition education have, too, been reported to sufficiently enhance the specific components of MetS in the community, which supports the generalizability of dietary interventions in the decrease of metabolic risk (11). This literature is expanded as the current results show that these improvements can be attained in older adults with routine clinical nutritional interventions.

The discovery of the main predictors of metabolic syndrome improvement gives an understanding of the factors that were most closely related to the positive results in the geriatric population. Compliance with a Mediterranean-type diet, increased fiber consumption, and at least 5% weight loss proved to be the most significant independent predictors, and that the quality of dietary patterns and a slight decrease in weight play an essential role in metabolic recovery. These results are in line with the new evidence on personalized and pattern-based nutrition strategies which combines bioactive compounds, macronutrient homeostasis, and individual metabolic profiles to maximize cardio-metabolic health (32). The protective relationships that have been found to exist with omega-3 supplementation, vitamin D deficiency, and low sodium intake are consistent with findings that show that nutrient modulation is possible to do with the aim of improving metabolic flexibility and lowering cardio-metabolic risk (33). The effect of physical activity also highlights the synergistic action of diet and lifestyle behaviors whereas the association of old age and more severe metabolic during baseline further indicates the cumulative weight of metabolism with age. Taken together, these results endorse comprehensive diet-based programs to address metabolic syndrome in the elderly, which strengthens the significance of the organized nutritional interventions as the foundation of cardio-metabolic risk mitigation.

### 4.1 Strengths of the study

- The study is large with a long-term follow-up, which increases the power of the statistical tests and the possibility to measure the long-term trends in metabolism among older adults.
- There is the high level of external validity that is supported by the use of real-world clinical data compared with the situation of tight control that is characteristic of laboratory studies.
- The overall evaluation of the metabolic syndrome components such as anthropometric, glycemic, lipid, blood pressure, inflammatory, oxidative stress, and dietary criteria provide a combined picture of the metabolic health modifications.
- The multivariate analyses facilitated the process of determining independent factors that predicted improvements in the MetS, which justified clinical and practical implications.

### 4.2 Limitations of the study

- The retrospective observational type does not provide the opportunity to make any causal inference and is exposed to residual confounding.
- Adherence and dietary intake were determined by clinical documentation as opposed to standardized dietary assessment scales, which creates the possible bias of misclassification.
- Subsets of participants only had inflammatory and dietary biomarker data, and this could restrict the generalizability of these results.
- The intervention intensity, duration and practices of the healthcare providers could not be completely standardized.
- The physical activity was also moderated but not objectively measured, which can underestimate the interactions of the physical activity with dietary interventions.

### 4.3 Future directions

- Prospective, pragmatic studies incorporating standardized dietary assessment instruments and yet maintaining a practical application should be incorporated in future studies.
- There is a need to conduct longitudinal studies on the maintenance of dietary change and its effect on functional outcomes, frailty, sarcopenia, and quality of life in elderly people.
- Individualized nutrition strategies that include metabolic phenotyping, comorbidity patterns and medication intake can further streamline the management of MetS.
- The development of mixed diets, behavioral, and digital health interventions may increase the adherence and long-term outcomes.

## 5 Conclusion

This study shows that the nutritional interventions applied in daily clinical care are connected with the improvement of the risk factors of metabolic syndrome in older people significantly. The relevant improvements in central obesity, dysglycemia, dyslipidemia, hypertension, and systemic inflammation were converted into significant levels of MetS resolution. These results strengthen nutrition-based interventions as non-pharmacological core health interventions to managing metabolic syndrome and enhance their full inclusion in geriatric and chronic disease care pathways to foster healthy aging.

## Data Availability

The raw data supporting the conclusions of this article will be made available by the authors, without undue reservation.
